# Analysis of Microplastics in Takeaway Food Containers in China Using FPA-FTIR Whole Filter Analysis

**DOI:** 10.3390/molecules27092646

**Published:** 2022-04-20

**Authors:** Xuejun Zhou, Jin Wang, Jiefang Ren

**Affiliations:** Zhejiang Institute of Product Quality and Safety Science, Hangzhou 310018, China; wangj@zjzay.com (J.W.); renjf@zjzay.com (J.R.)

**Keywords:** microplastic, takeaway food containers, database matching, micro-FT-IR imaging, human health

## Abstract

With the rapid development and popularization of the internet and smartphone industry for ordering and delivery, the consumption of takeaway food is increasing globally, especially in China. However, there is little information about microplastics in takeaway food containers, so their potential risks to human health remain unknown. This study explored the possibility of using focal plane array (FPA)-based micro-FT-IR imaging to detect microplastics released from food containers and evaluated their contents using an automated database matching analysis method. We investigated microplastics in seven types of food containers widely used in China. The most common plastic types observed were polyamide (PA), polyurethane (PU) and polystyrene (PS), which were found to comprise 22.8%, 18.2%, and 8.5% (number of particles) of all microplastics, respectively. Microplastics were found in all seven types of food containers, and the content excluding cellulose was 29–552 items/container. Our research shows that microplastics in takeaway food containers might originate from atmospheric sediment or flakes from the inside surface of the container. According to the content of microplastics in takeaway food containers, people who order takeaway food 5–10 times a month might consume 145–5520 microplastic pieces from food containers.

## 1. Introduction

Due to the rapid development and popularity of the internet and smartphone industries, the consumption of fast food for ordering and delivery is rapidly increasing [1]. According to data from iResearch (2019) [2], the takeaway food market of China was worth 653.6 billion yuan in 2019, an estimated increase of 39.3% compared to 2018, and there are no signs of it slowing down [3,4]. Meituan and ele.me have grown into two well-known and increasingly popular online food delivery service platforms. Each of these companies is reported to deliver 30 million orders every day. In recent years, with the successive introduction and implementation of policies such as the “Outline of the Healthy China 2030 Plan” and “The State Council’s Opinions on Implementing the Healthy China Action” (The State Council, the People’s Republic of China, 2016 and 2019), health has become a national-level strategy. The food industry plays an important role in maintaining human health. To date, white-collar workers have consumed a large amount of takeaway food every day. Increasing takeaway food consumption has caused various sustainability issues, including environmental sustainability issues and food safety.

Takeaway food containers are commonly made of petroleum-based plastics, which offer a number of advantages, including their low cost, light weight, good processability and high performance. With the strong development of the delivery and takeaway food market, disposable food packaging produces a large amount of plastic waste. It is estimated that in 2017 alone nearly 40 million disposable plastic products were used and dumped every day in China (e-Marketer 2018) [5]. Plastic fragments are formed into macro, micro, and nanoparticles through various physical, chemical and biodegradation processes, including mechanical abrasion, embrittlement, temperature changes, photooxidation and other effects [6]. This issue leads to an increased and wider proliferation and accumulation of microplastics (MPs) and nanoplastics (NPs) in the environment. Therefore, a series of national initiatives to reduce the use of plastics has been implemented, with further measures in preparation. In 2020, the NDRC (National Development and Reform Commission, 2020) and MEE (Ministry of Ecology and Environment, 2020) [7] of the Chinese government issued “Opinions on Further Strengthening the Prevention and Control of Plastic Pollution”. “Opinions” proposed that, by the end of 2022, the consumption of disposable plastic products should be greatly reduced, and alternative products should be promoted.

To date, many investigations have shown that microplastics can be released from food containers into food and pose a potential health threat due to the decomposition of polymers. According to reports, billions of microparticles and nanoparticles can be released into tea from plastic tea bags [8]. Fadare et al. reported that humans are exposed to 188 tons of microplastics each year [9]. Du et al. [10] suggested that 3–29 microplastic pieces could be released by take-out containers. In addition, a recent study estimated that up to 16,200,000 particles/L microplastics could be released during the preparation of infant formula in PP baby feeding bottles [11]. Microplastics have been reported to penetrate organs, tissues or cells [12,13,14], which can worsen the toxicological and pathological responses of organisms, leading to increased oxidative stress [15,16], inflammation [17], and metabolic disorders [18,19,20]. In the context of plastic food packaging, takeaway food containers are widely used by people for the storage, rapid heating and transportation of take-out food orders. These can be a source of human exposure to microplastics, especially given their poor heat resistance. However, little information is available on microplastics in takeaway food containers, and there are no specific regulatory limits for particle migration from food contact materials, so their risks to human health remain unknown. Robust standardized analysis methods for sampling, processing, and analysis remain lacking.

Hence, we systematically investigated seven types of food containers that are widely used in China, including polypropylene, polyethylene, polyethylene terephthalate and nylon. A combined approach utilizing focal plane array-based micro-Fourier transform infrared microscopy and scanning electron microscopy was used to quantitatively study the microplastics in takeaway food containers. The content of microplastics was evaluated using a database matching method. Finally, we assessed the potential risks of human exposure to these microplastics based on the abundance of microplastics in food containers and the ordering frequency of white-collar workers. The results show that the correlation coefficient method is a potential tool to detect and characterize microplastics in food containers. The proposed methods provide an effective automated approach to characterize and calculate the abundance of microplastic polymers and have substantial benefits for standardizing this procedure.

## 2. Materials and Methods

### 2.1. Sample Collection

**Figure 1:**
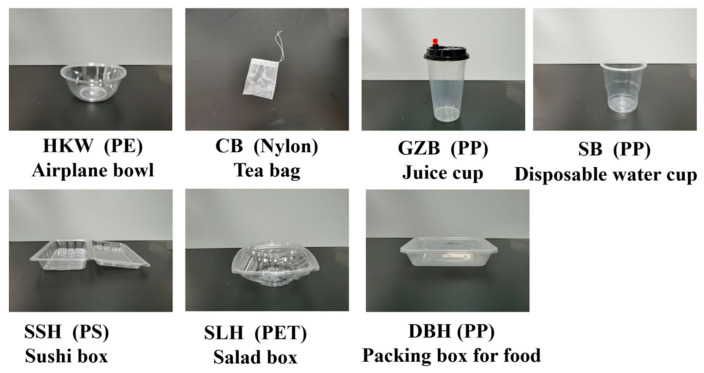
Seven types of takeaway food container.

Seven types of widely used food containers were purchased from local supermarkets in Hangzhou. These containers, commonly referred to as “take away packs”, are known to be used for quick food delivery and packaging, and come sealed in 20–50 piece packages. The teabags analyzed contained 10 pieces per pack. Information regarding these plastic containers was collected from the packaging labels (Figure 1).

### 2.2. Sample Preparation

Prior to the formal experiment, a preliminary experiment was conducted to simulate the use of food packaging materials in real life. To avoid background contamination in the lab, throughout the experiment, the researchers wore only clean lab cotton coats and nitrile gloves. All samples were processed on a laminar flow bench to reduce airborne contamination during the extraction process. In order to minimize MP contamination deriving from the equipment used for sample preparation, all lab tools were flushed with filtered (1.2 µm) Milli-Q water three times before use. CB (nylon) containers were steeped in ultrapure water at 100 °C for 30 min, while the other food containers were directly rinsed with ultrapure water and subsequently shaken for 5 min in a shaker at 160 rpm, followed by steeping for 1 h. All samples were covered with lids during processing. The volume of ultrapure water added depended on the size of the container (100–500 mL per container). The main criterion for the amount of ultrapure water added was that it should completely cover the bottom of the food container or sufficiently cover the surface of the plastic bag. Then, under laboratory conditions, a gold-plated polycarbonate membrane (pore size 0.4 μm, diameter 25 mm) was used to filter the extracted suspension water of each sample through a vacuum pump at a pressure of about 0.1 bar (SHZ-D, Lichen, Shanghai, China). The experiment for each type of food container for each treatment was repeated three times (one filter per sample). After filtration, the filter membranes were carefully transferred to glass dishes with lids, dried at room temperature, and stored for future analysis.

Control groups (glass beakers) were also treated in the same way to correct for any potential procedural contamination. Both samples and control groups were covered with lids when not being processed.

### 2.3. Characterization of Microparticles

The chemical compositions of the particles were identified by using focal plane array (FPA)-based micro-FT-IR imaging on a Nicolet IN10 MX Fourier transform infrared (FT/IR) spectrometer equipped with an FPA/mercury–cadmium–telluride (MCT) imaging detector. A background scan was collected before each sample scan on a clean window in the range of 4000–700 cm^−1^ with an 8 cm^−1^ spectral resolution applying 64 co-added scans in reflectance mode and a pixel size of 25 µm. An area of 8 × 8 tiles was scanned on the sample window following the background scan with the same parameters applying 64 co-added scans per pixel. In the study, the scan of each whole filter was divided into four zones with the same parameters; all four area images were then combined to construct a pseudocolor image to record the spectra of all deposited particles on the filter. All samples and control groups were measured with the μ-FTIR-FPA method using the same parameters.

The morphology and structure were studied with a scanning electron microscope (JEOL JSM 6510). The elemental distribution of the samples was determined using an Oxford INCA Energy 350 energy-dispersive X-ray spectrometer equipped with SEM.

### 2.4. Data Analysis

Statistical analysis was performed using MATLAB R2014a. After loading the data and reference spectra (Thermo Scientific infrared spectrum library database), the spectral fit between the two was calculated by Pearson correlation for the untreated data. The MATLAB software identifies the recorded spectra based on the results of the Pearson correlation factors (r) calculated for the respective characteristic spectra. Spectra are counted as identified only if the r value is higher than 0.7 (70% of the match quality metrics are considered acceptable) assigned to the same polymer entry, and the polymer type is added to the list of analyzed pixels together with the (x,y) coordinates. The detailed steps are as follows: first, we selected the characteristic microplastic spectral regions to be 717–2000 cm^−1^; then, we set the Pearson correlation factor value of 0.7 to extract the isolated singular points in the image; finally, we calculated the Pearson correlation coefficient between the spectrum of each pixel point and the standard spectrum one by one. In this report, the number of pixels was considered to represent the number of microplastics we found.

## 3. Results

### 3.1. Abundances of Microplastics

**Figure 2:**
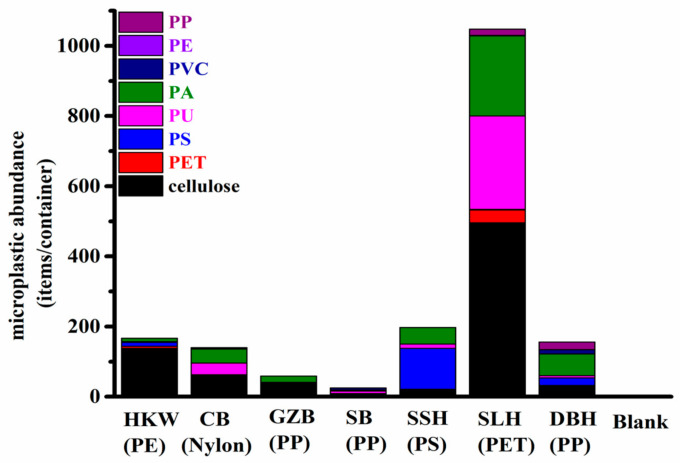
Microplastic abundance in take-out containers (items/container).

**Figure 3:**
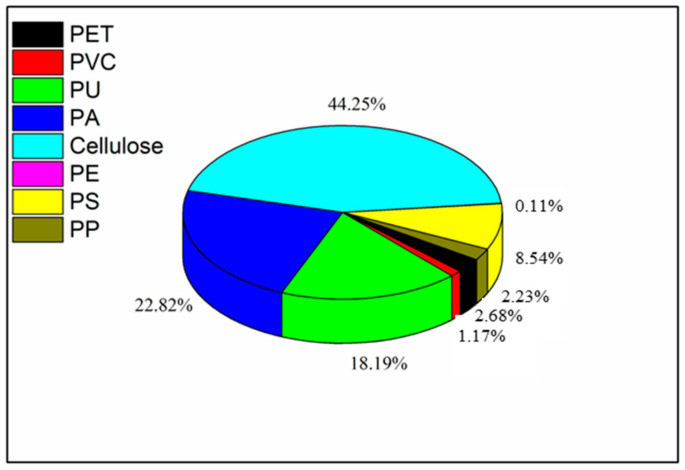
Polymer content of microplastics from takeaway food containers.

Seven types of commonly used takeaway food containers were analyzed for the amount of microplastics they contained by using μ-FTIR-FPA, and the results are shown in Figure 2 and Figure 3. In total, 1792 suspected particles were identified. With the exception of cellulose, the content of microplastics contained in the takeaway food containers was 29–552 items/container. Individual contents significantly varied with the container material, and the highest content was observed in SLH (PET) containers. The most common types of plastics observed were cellulose, polyamide (PA), polyurethane (PU) and polystyrene (PS), which together accounted for 44.3%, 22.8%, 18.2% and 8.5% (number of particles) of all microplastics, respectively. In addition, other types of microplastics were discovered, comprising PET (polyethylene terephthalate), PP (polypropylene), PVC (polyvinyl chloride) and PE (polyethylene), with proportions of 2.7%, 2.2%, 1.2% and 0.1%, respectively. The detailed chemical composition of microplastics contained in takeaway food containers is shown in Figures S1 and S2. Among them, some have identical compositions to the original containers (PA, PS, PP, PE and PET). Therefore, these are considered to be microplastics that have peeled from the takeaway containers. Other types of microplastics (cellulose, PU and PVC) are believed to have been introduced from another source. The percentage of same/other polymer microplastics for each container are presented in Table 1. The differences in microplastic abundances among the different types of take-out containers may be associated with different material characteristics caused by different manufacturing processes. The manufacturing processes of PET and PP containers involve the pressurized injection of a melted masterbatch into a mold cavity, resulting in smooth surfaces, while the PS containers are formed by injecting gas into a melted PS masterbatch, which results in a relatively loose structure. Taking the microplastic abundance, manufacturing process and surface characteristics of different types of containers into consideration, we speculate that the loose structure may cause microplastics to flake from the inner surface more easily. For reference, Du et al. (2020) also found that PS containers generated mostly PS particles. Given the large amount of cellulose- and PA-type microplastics, we assume that this contamination may come from atmospheric fallout during the production, storage and transportation of takeaway food containers. Several studies [21] show that synthetic textiles are the main source of microplastics in the air, occurring mostly as fibers. It has been determined that 29% of these fibers are composed of synthetic material.

### 3.2. Size and Shape Distribution of Microplastics

**Figure 4:**
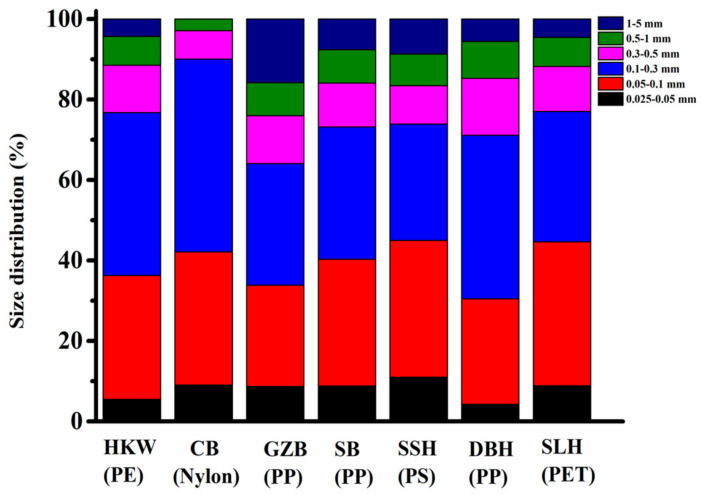
The size distribution of microplastics found in takeaway containers. The particles are divided into 6 size ranges: 0.025–0.05 mm, 0.05–0.1 mm, 0.1–0.3 mm, 0.3–0.5 mm, 0.5–1 mm and 1–5 mm.

The distribution of MP size from takeaway food containers shows that although some microplastics are larger in size, most microplastics have a smaller size range. The detection limit of the equipment was 25 μm and the pore size of the filter used was 0.4 μm. Therefore, we divided microplastics into six categories (0.025–0.05 mm, 0.05–0.1 mm, 0.1–0.3 mm, 0.3–0.5 mm, 0.5–1 mm and 1–5 mm) and two types (fibers and fragments). As shown in Figure 4, the lengths of most microplastics in PE containers (HKW) are 0.05–0.1 mm and 0.1–0.3 mm, accounting for 30.8% and 40.5% of the total number of particles, respectively, followed by 0.3–0.5 mm (11.7%). Furthermore, particle size distributions (PSDs) of 1–5 mm and 0.025–0.05 mm were found in PE containers, accounting for 4.3% and 5.5% of particles, respectively. The particle size distribution ratios of different microplastics in the seven types of containers were similar. The size distribution histogram of all samples together is presented in Supplementary Materials Figure S3.

The distribution of the MP forms released from takeaway food containers is shown in Figure S4. The types of microplastics in the seven containers varied. The most common type of MP observed in PE containers (HKW) was fiber (82.6%), whereas the proportion of fiber in PS containers (SSH) was 10.2%. For nylon (CB) samples, approximately 43% and 57% of microplastics were of fiber and fragment types, respectively.

### 3.3. Characteristics of Microplastics

**Figure 5:**
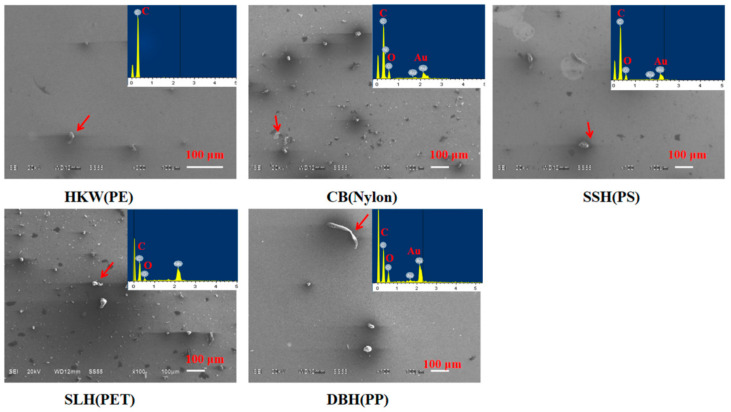
SEM Figures of microplastics contained in takeaway food containers.

SEM-EDS was employed to observe the morphology and elemental composition signatures of the plastic particles from the surface of the takeaway food containers. Figure 5 shows different abundances of plastic particles on the surface of each container, which can be easily detached through a direct flushing treatment. Various sizes and shapes were observed in all five types of containers. The shapes of the observed particles include fibers, irregular fragments and spheres. All microplastics exhibit the characteristics of surface roughness and edge traction. The corresponding EDS spectrum of microplastics displayed significant carbon and oxygen peaks, which were further confirmed to be cellulose, PET, PS, PP and PE plastic following FT-IR analysis.

### 3.4. FPA-Based Micro-FT-IR Validation and Data Analysis of Takeaway Food Containers

**Figure 6:**
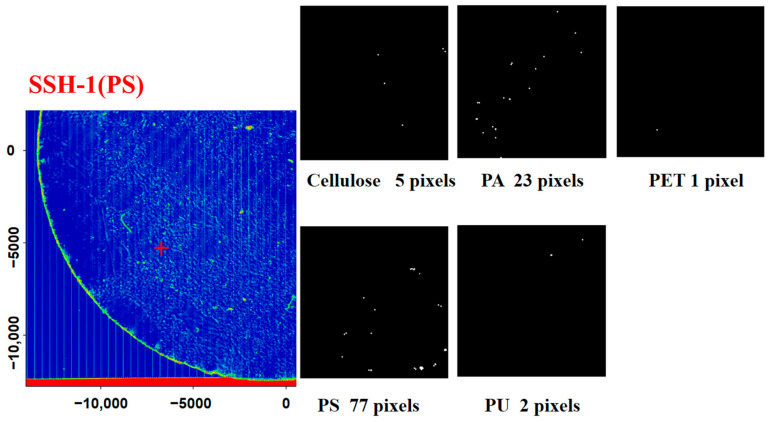
Microplastic abundance in the SSH(PS) sample (quarter part).

**Figure 7:**
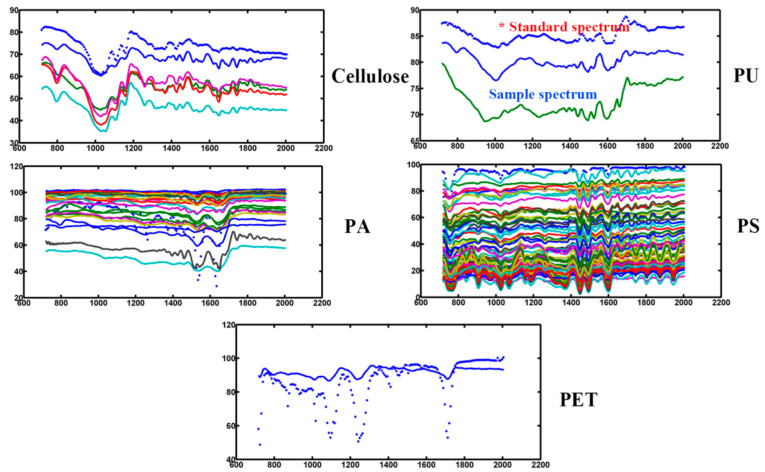
FTIR spectra of typical selected microplastic particle samples in the region of 700–2000 cm^−1^.

In contrast to these single-point measurements, the coupling of the FPA detector enables an image of the entire filter area. This type of analysis has the advantage of simultaneously collecting chemical (spectral) and spatial information of several particles by automated mapping of a sample, which enables the analysis of small microplastics without manual sorting and the estimation of particle features such as their areas and diameters [22,23,24,25]. In this study, the scan of each whole filter was divided into four zones with a spatial resolution of 25 μm × 25 μm. We used identical parameters to image each area and subsequently combined all four area images to construct a pseudocolor image. Then, we imported the spectral data into the MATLAB R2014a software to calculate the microplastics in takeaway food containers. Taking the results of the SSH(PS) sample as a representative (Figure 6), we can see that five pixels of cellulose, 23 pixels of PA, one pixel of PET, 77 pixels of PS and two pixels of PU can be detected in the picture. For the SSH(PS) sample, the microplastics and standard plastic material have similar FTIR absorptions with detection of the same characteristic peaks at 717–2000 cm^−1^ (Figure 7). The absorption bands at 1730 cm^−1^ and 1640 cm^−1^ correspond to the typical carbonyl absorption of (ν C=O), and the peaks at 1550 cm^−1^, 1260 cm^−1^ and 1130 cm^−1^ are related to the stretching vibration of (ν C-N) and (ν C-O). The observed peaks at 1601 cm^−1^ and 1492 cm^−1^ can be associated with benzene ring skeleton vibration (δ C=C), while the observed peaks at 695 cm^−1^ and 755 cm^−1^ indicate the hydrocarbon group of unsaturation (δ=C-H) in the benzene ring caused by vibration of curvature outside the plane; the peak at 1452 cm^−1^ can be attributed to the symmetrical vibration of the curvature of the methylene group (δs CH_2_) [26,27]; Figures S5–S11 show the false color images of the entire filter membrane for the seven takeaway food container samples with the entire 25 mm filter membrane. Taking the SSH(PS) as a representative, we can see that five types of plastic polymers (cellulose, PET, PS, PU, PA) were discovered, and their content was 20, 1, 117, 12 and 47 items/container, respectively.

### 3.5. Estimation of Microplastic Intake by Humans

Human swallowing of microplastics via takeaway food containers was studied and subsequently calculated based on the average abundance of microplastics in takeaway containers and data on takeaway ordering frequency. According to data from the Meituan Research Institute, by 2020, the number of Chinese takeaway customers had reached 456 million, and the penetration rate of Chinese food and beverage delivery had reached 96.31%. In addition to traditional meals, afternoon tea and supper have become new favorites for consumers to order. The average number of takeaway food orders by white-collar workers in 2021 in China is 5–10 times monthly.

The abundance of microplastics in takeaway food containers and glass bakers is presented in Table 2. Microplastics were found in all takeaway containers, and the content excluding cellulose was 29–552 items/container. In contrast, a small number of microplastics were found in the control groups (glass beaker). Cellulose was the main type, accounting for nearly 100% of the total microplastics. In the present study, air quality was strictly controlled during the experiments. However, air contamination is extremely hard to eliminate even in strictly controlled conditions.

Based on the microplastic abundance in takeaway food containers, people who order takeaway food 5–10 times monthly may ingest 145–5520 pieces of microplastics from containers. This estimation is based on an average of 3.27 plastic items per container (e.g., boxes, bags, pieces of shrink wrap), and assumes that only one container is used for each order. These assumptions, as well as the detection limitations (25 μm) of the instruments, may result in an underestimation of the human intake of microplastics from these sources.

## 4. Conclusions

With the rapid development and popularization of the internet and smartphone industry for ordering and delivery, the consumption of takeaway food is increasing worldwide, particularly in China. Understanding the distribution and abundance of microplastic contamination can help us to examine how microplastics migrate from food contact materials, identify which plastic items will affect contamination and take steps to set specific regulatory restrictions on food contact materials.

In the present study, we quantitatively investigated microplastics in takeaway containers using a focal plane array-based micro-Fourier transform infrared microscope and evaluated their content using the Pearson correlation coefficient method. We proved that commercial victual packaging materials can release microplastics during daily usage, which is indeed a matter worthy of public concern. As a result, microplastics were found in all takeaway containers, and the content excluding cellulose was 29–552 items/container. In contrast, a small number of microplastics was found in the control groups. There were two major sources of microplastics in takeaway containers: atmospheric fallout and particles flaking from the inner surface of containers. Based on the microplastic abundance in takeaway containers, people who order takeaway food 5–10 times monthly may ingest 145–5520 pieces of microplastics from these containers. The results show that the correlation coefficient method is a feasible tool to detect and characterize microplastics in takeaway food containers.

## Figures and Tables

**Table 1:** The percentage of same/other polymer microplastics for seven takeaway food containers.

Sample	Same Microplastics Percentage (%)	Other Polymer Microplastics Percentage (%)
HKW(PE)	PE (0.00)	100
CB(Nylon)	Nylon (28.57)	71.43
GZB(PP)	PP (0.00)	100
SB(PP)	PP (0.00)	100
SSH(PS)	PS (59.39)	40.61
SLH(PET)	PET (3.44)	96.56
DBH(PP)	PP (14.1)	85.9

**Table 2:** Microplastic abundance in take-out containers and glass bakers.

Sample	Microplastic Abundance (Items/Container)	Sample	Microplastic Abundance (Items/Container)
HKW(PE)	Cellulose 138, PA 10, PET 6, PS 11, PU 2	Glass beaker	Cellulose 6
CB(Nylon)	Cellulose 61, PA 40, PET 2, PS 2, PE 2, PVC 2, PU 33	Glass beaker	Cellulose 4
GZB(PP)	Cellulose 38, PA 18, PET 3	Glass beaker	Not found
SB(PP)	Cellulose 8, PA 3, PVC 6, PS 1, PU 7	Glass beaker	Not found
SSH(PS)	Cellulose 20, PA 47, PET 1, PS117, PU12	Glass beaker	Cellulose 4
SLH(PET)	Cellulose 496, PA 229, PET 36,PS 2,PU 266,PVC 1, PP 18	Glass beaker	Cellulose 12, PA 5
DBH(PP)	Cellulose 32, PA 62, PS 12, PU 6, PVC 12, PP 22	Glass beaker	Cellulose 5

## Data Availability

Not applicable.

## Supplementary Materials

The following supporting information can be downloaded at: https://www.mdpi.com/article/10.3390/molecules27092646/s1. Figure S1: Chemical compositions of microplastics from takeaway food containers (HKW, CB, GZB SB); Figure S2: Chemical compositions of microplastics from takeaway food containers (SSH, SLH, DBH); Figure S3: Size distribution histogram of all samples together; Figure S4: Distribution of microplastic types from takeaway food containers.; Figures S5–S11: False color images of the entire membrane filter of the seven takeaway food container samples.

## Sample Availability

Samples of the compounds are available from the authors.
