# Supplementary material for: Analysis of Microplastics in Takeaway Food Containers in China Using FPA-FTIR Whole Filter Analysis
Source: Molecules. 2022 Apr 20;27(9):2646. doi: 10.3390/molecules27092646 (PMC9103929; doi:10.3390/molecules27092646)
Supplement: Supplementary file 1 [file molecules-27-02646-s001.zip › molecules-1683739-supplementary.pdf]

Supporting information

Microplastic in takeaway food containers in China using FPA-FTIR whole filter analysis

Xue-jun Zhou*, Jin Wang, Jie-Fang Ren

Zhe Jiang Institute of Product Quality and Safety Science, Hangzhou, Zhe Jiang, 310018, China.

E-mail: 23554243@qq.com Telephone: 86-0571-85798712

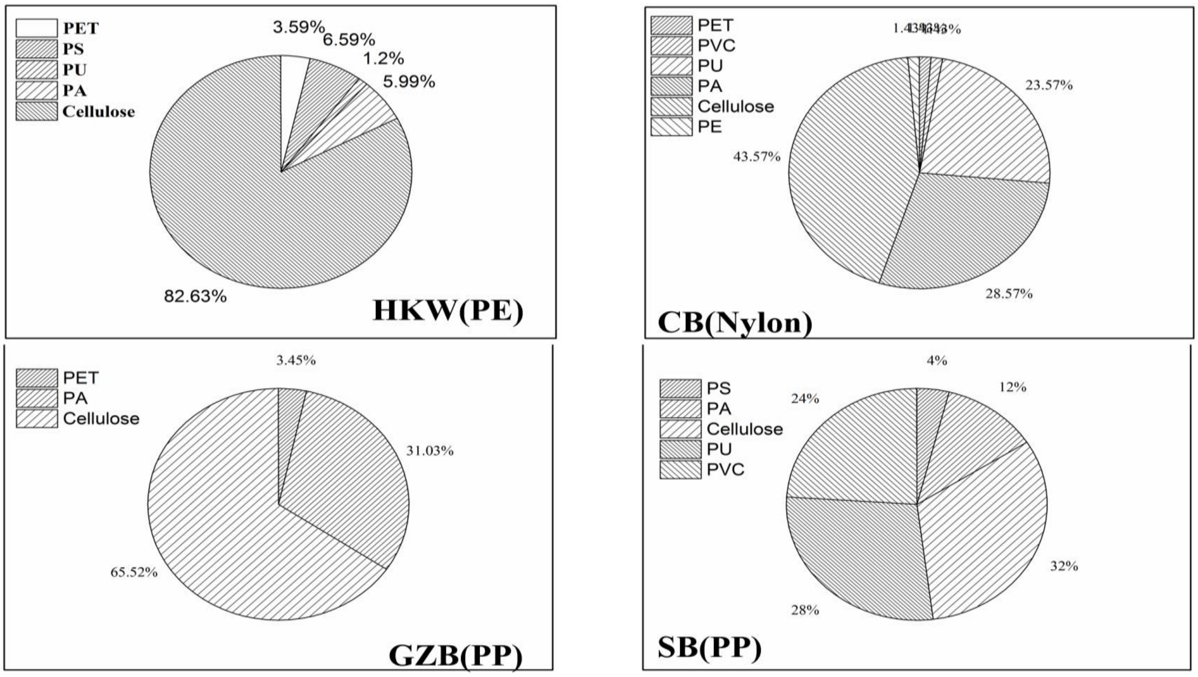

Figure S1. Chemical compositions of microplastics from takeaway food containers (HKW, CB, GZB SB).

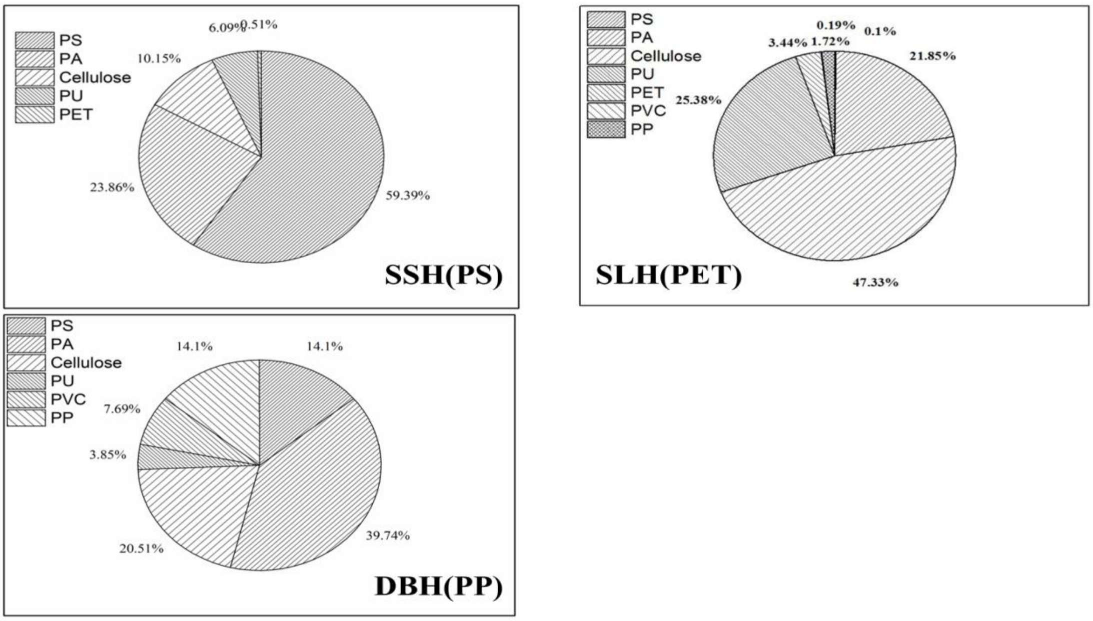

Figure S2. Chemical compositions of microplastics from takeaway food containers (SSH, SLH, DBH).

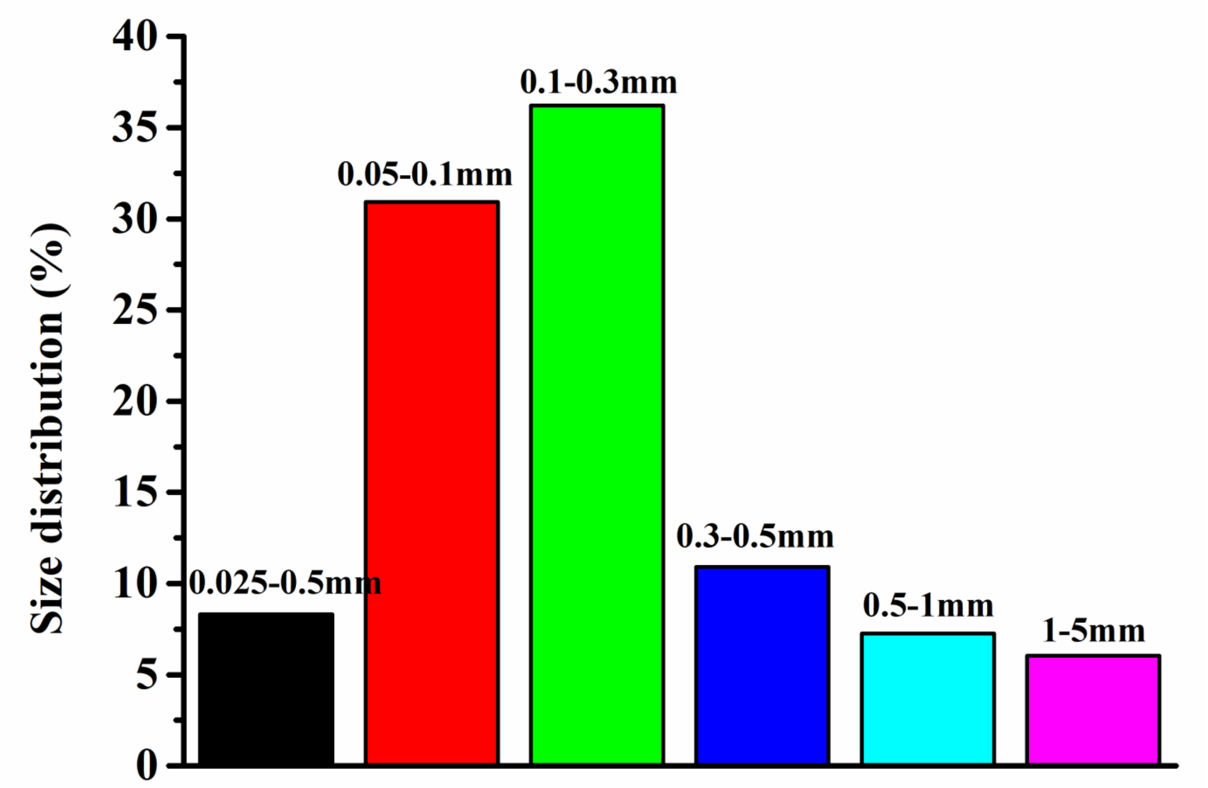

Figure S3. Size distribution histogram of all samples together

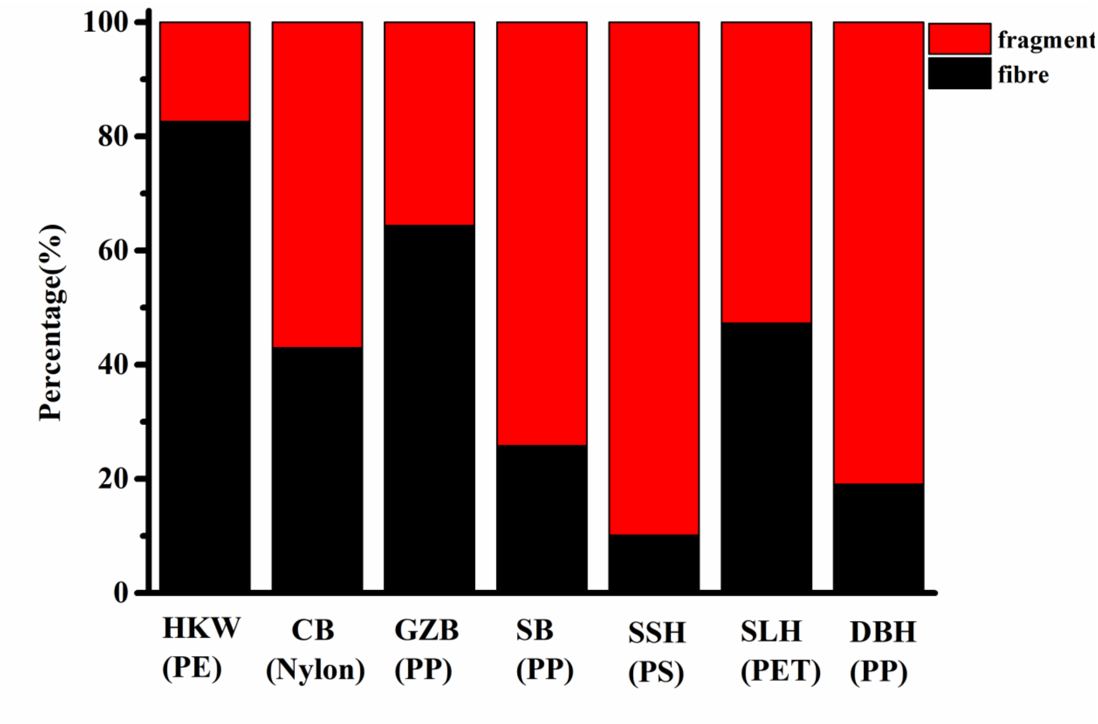

Figure S4. Distribution of microplastic types from takeaway food containers.

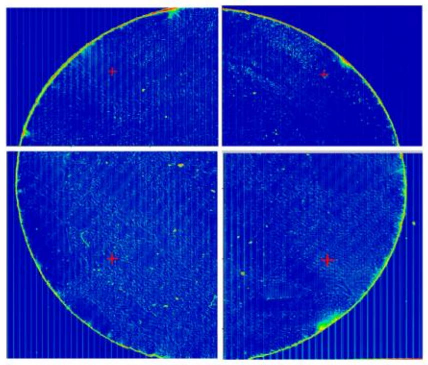

sample	cellulose	PET	PS	PU	PA
SSH-1	5	1	77	2	23
SSH-2	9	0	8	4	10
SSH-3	2	0	23	5	14
SSH-4	4	0	9	1	0
Total	20	1	117	12	47

Figure S5. False color images of the entire membrane filter of an SSH(PS) sample.

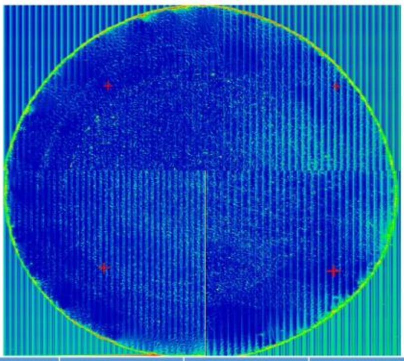

Sample	Cellulose	PS	PA	PE	PET	PVC	PU
CB-1	2	2	25	0	0	0	0
CB-2	12	0	0	2	2	2	21
CB-3	1	0	4	0	0	0	8
CB-4	46	0	11	0	0	0	4
Total	61	2	40	2	2	2	33

Figure S6. False color images of the entire membrane filter of an CB(Nylon) sample.

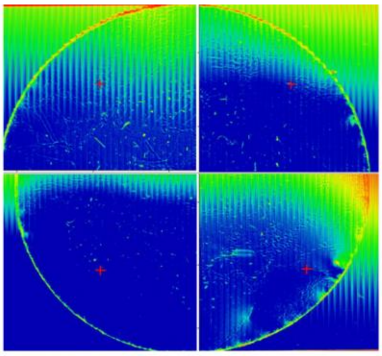

Sample	Cellulose	PS	PA	PP	PVC	PU
DBH-1	4	0	40	0	11	2
DBH-2	2	0	17	0	0	3
DBH-3	23	12	0	22	1	1
DBH-4	3	0	3	0	0	0
Total	32	12	62	22	12	6

Figure S7. False color images of the entire membrane filter of an DBH(PP) sample.

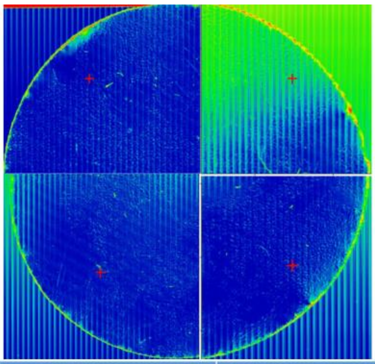

Sample	Cellulose	PA	PET
GZB-1	6	4	0
GZB-2	19	10	2
GZB-3	2	3	1
GZB-4	11	1	0
Total	38	18	3

Figure S8. False color images of the entire membrane filter of an GZB(PP) sample.

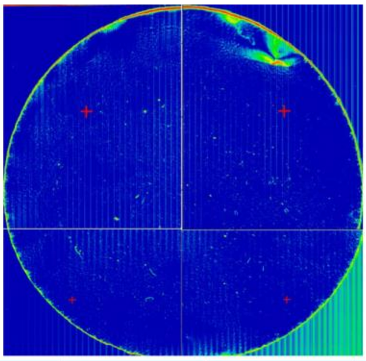

Sample	Cellulose	PA	PET	PS	PU
HKW-1	19	0	0	2	1
HKW-2	51	3	6	0	1
HKW-3	29	0	0	3	0
HKW-4	39	7	0	6	0
Total	138	10	6	11	2

Figure S9. False color images of the entire membrane filter of an HKW(PE) sample.

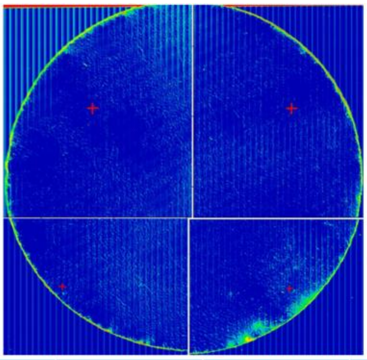

Sample	Cellulose	PA	PVC	PS	PU
SB-1	0	0	0	0	3
SB-2	5	0	6	0	0
SB-3	0	0	0	1	1
SB-4	3	3	0	0	3
Total	8	3	6	1	7

Figure S10. False color images of the entire membrane filter of an SB(PP) sample.

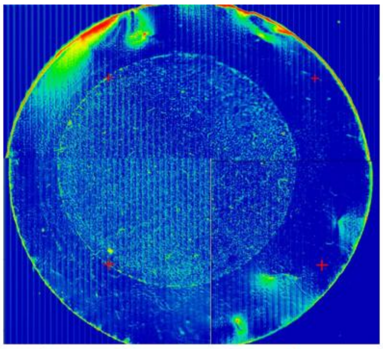

Sample	Cellulose	PET	PVC	PS	PU	PA	PP
SB-1	245	23	0	1	121	56	18
SB-2	134	0	0	0	0	91	0
SB-3	35	2	0	1	51	15	0
SB-4	82	11	1	0	94	67	0
Total	496	36	1	2	266	229	18

Figure S11. False color images of the entire membrane filter of an SLH(PET) sample.
